# Hyaluronic acid filler injection for upper body aesthetic contouring

**DOI:** 10.1016/j.jpra.2025.07.006

**Published:** 2025-07-23

**Authors:** Kyu-Ho Yi, Jovian Wan, Song Eun Yoon, Lucas Basmage, Carlos Bautzer

**Affiliations:** a Division in Anatomy and Developmental Biology, Department of Oral Biology, Human Identification Research Institute, BK21 FOUR Project, Yonsei University College of Dentistry, Seodaemun-gu, 03722, Korea; b You and I Clinic, Seoul, Korea; c Medical Research Inc., Wonju, Korea; d BRANDNEW Aesthetic Surgery Clinic, Seoul, Korea; e Synergie Clinic, Campo Grande, Brazil; f Lifestyle Clinic, Sao Paulo, Brazil

**Keywords:** Hyaluronic acid filler, Higher BMI contouring, Subcutaneous adiposity, Nonsurgical pectoral lift, Male aesthetic refinement

## Abstract

The demand for non-surgical upper body enhancement continues to grow, particularly among men seeking improved muscular definition without the risks of surgery or functional compromise. This case report presents a novel protocol using high G-prime hyaluronic acid (HA) fillers (Sedifill; Maypharm, Seoul, Republic of Korea) to contour the pectoralis major, deltoid, biceps, and brachioradialis in a 41-year-old male with moderate subcutaneous adiposity (BMI: 25 kg/m²; body fat: 21 %). A total of 134 mL of filler was injected subcutaneously using a layered technique with dynamic assessment and feathering to enhance muscle visibility while integrating with surrounding fat. Immediate improvements were noted in chest lift, deltoid rounding, and arm tapering, with no complications at one-month follow-up. The patient reported high satisfaction and increased confidence in appearance. This approach demonstrates the feasibility and adaptability of HA filler contouring in individuals with higher body fat, highlighting the importance of customized protocols to overcome adipose masking and achieve natural, balanced results. While not a substitute for fat reduction, this technique offers a safe, reversible alternative for patients seeking aesthetic enhancement and may serve as a motivational catalyst for healthier lifestyle changes. Further studies are needed to assess long-term outcomes and validate efficacy across broader populations.

## Introduction

The pursuit of a sculpted upper body—characterized by defined pectorals, deltoids, and arm musculature—remains a central focus in contemporary male aesthetics.^1^, ^2^, ^3^ Previous studies have demonstrated the safety and efficacy of high-volume hyaluronic acid filler use in breast and buttock augmentation, providing a foundational rationale for its application in upper body contouring. While surgical implants can effectively enhance muscular contours, they are associated with significant risks, including infection, capsular contracture, and extended recovery periods.^4^, ^5^ Neuromodulators offer a less invasive alternative but achieve results through muscle atrophy, which may compromise spinal stability and functional strength over time. Additionally, conventional resistance training and anabolic supplementation often produce variable outcomes, particularly in individuals with genetic limitations to muscle hypertrophy or those unable to adhere to intensive exercise regimens.

Hyaluronic acid (HA) fillers, commonly used in facial volumization and collagen stimulation, represent a promising but underutilized modality for body contouring. Their viscoelasticity supports structural enhancement, while their biodegradability offers a key advantage in reversibility over permanent implants. This article introduces a novel protocol for upper body contouring using HA fillers, targeting the pectoral, deltoid, and upper extremity musculature, while similar uses have been reported, this case uniquely targets the deltoid and upper arm in an Asian male using a defined protocol. Through a single-case demonstration, we highlight the technique’s safety, efficacy, and relevance to evolving standards in male aesthetic refinement.

## Innovative ideas

**Figure 1:**
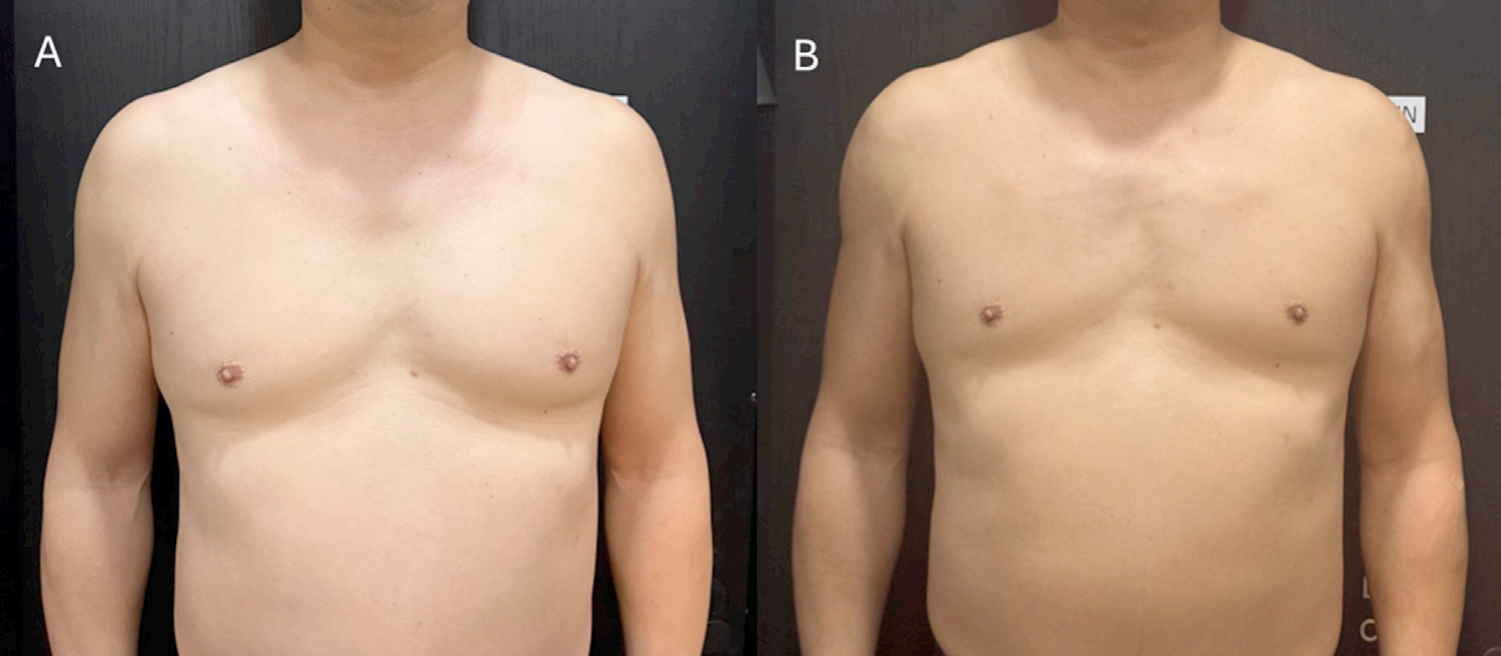
(A) Pre-treatment; (B) One-month follow-up, demonstrating enhanced upper body harmony and reduced adipose irregularity from the frontal view.

A 41-year-old male (BMI: 25 kg/m²; body fat: 21 %) presented with the goal of enhancing upper body muscular definition despite moderate subcutaneous adiposity (Figure 1A). The patient led a predominantly sedentary lifestyle, engaged in irregular resistance training, and sought a non-surgical approach to refine the contours of the pectoralis major, deltoids, and upper arms. His aesthetic objective was to create the illusion of underlying muscle structure rather than increased volume, acknowledging the limitations posed by his higher body fat percentage.

Although lower body fat is generally considered ideal for maximizing muscular definition, this case illustrates the adaptability of hyaluronic acid (HA) filler contouring in individuals with higher adiposity. A high G-prime HA filler (Sedifill; Maypharm, Seoul, Republic of Korea) was selected for its robust lifting capacity and ability to create shadowing effects, which contribute to perceived muscle definition. Filler was placed in the deep subcutaneous plane to camouflage superficial fat deposits and accentuate the natural topography of the underlying musculature. An 18-gauge, 70 mm blunt cannula was used, with injections delivered into the deep fat layer. Entry points were located at the origin of each targeted muscle.

**Figure 2:**
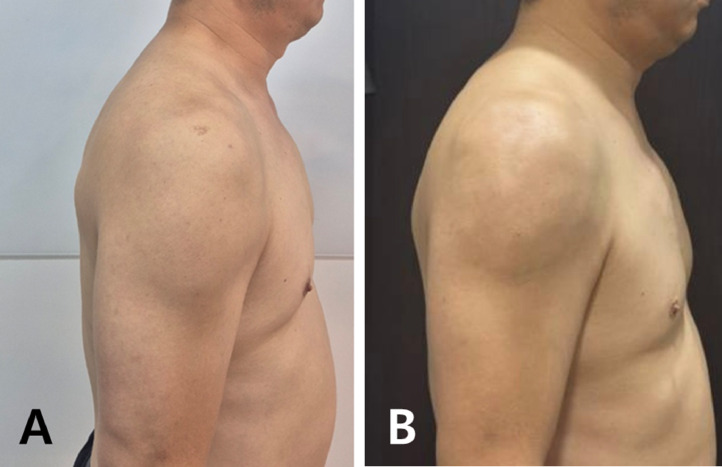
(A) Pre-treatment; (B) One-month follow-up, from the lateral view.

Immediately post-procedure, subtle yet perceptible enhancements were observed, including improved pectoral projection, deltoid rounding, and greater upper arm symmetry. At the one-month follow-up, filler integration had smoothed adipose irregularities and produced a more contoured, “toned” appearance despite no measurable change in the patient's body fat percentage (Figure 1B). The patient reported high satisfaction, rating the outcome as 8 out of 10, and specifically noted increased confidence when wearing fitted clothing (Figure 2).

## Pectoralis major augmentation

Injection volume was increased to 35 mL per side (from an initial 30 mL) to compensate for the masking effect of subcutaneous adiposity. The technique was optimized to include deeper subcutaneous placement along the inferomedial border of the pectoralis major, enhancing the natural chest crease and providing a subtle lifting effect. Particular emphasis was placed on defining the inframammary fold, which contributed to a more elevated and youthful chest contour, aligning with athletic male aesthetics.

## Deltoid refinement

For the deltoid region, a volume of 40 mL per side was used to avoid overprojection while achieving visible enhancement. A layered injection technique targeted both superficial and deep subcutaneous planes: superficial aliquots were employed to smooth out adipose irregularities, while deeper deposits simulated the septated architecture of the deltoid muscle. Feathering techniques were applied at the periphery of the filler to ensure a gradual transition into adjacent fat tissue, thereby minimizing visible demarcation.

## Biceps and brachioradialis enhancement

To improve upper limb definition, 12 mL of HA filler was injected per side into the biceps and brachioradialis regions. Linear threading along the medial biceps ridge and along the brachioradialis created a more pronounced taper from the upper arm to the forearm. This approach aimed to harmonize the silhouette while enhancing muscle visibility through moderate adiposity.

Dynamic assessment during injection was integral to ensuring that the enhancements appeared natural during limb movement. Adipose integration techniques were used throughout to blend the filler margins into surrounding soft tissue, preventing contour irregularities and optimizing overall aesthetic coherence.

## Discussion

A primary challenge in treating individuals with higher body fat percentages is adipose masking, wherein subcutaneous fat obscures the contouring effects of hyaluronic acid (HA) filler. This limitation necessitates increased filler volumes and strategic multi-plane layering to achieve visible definition. For example, in this case, augmenting the pectoralis major volume from 30 mL to 35 mL per side was critical to overcoming fat-induced masking and achieving appreciable chest enhancement. Similar volume and technique adjustments were required in the deltoid and biceps regions to maintain balance and natural proportionality. These adaptations underscore the importance of individualized treatment planning based on body composition rather than adherence to standardized protocols.

Patient expectation management is central to achieving high satisfaction. In patients with moderate to high adiposity, the primary objective is to create the *illusion* of muscularity rather than sharply defined musculature typically seen in lean individuals. This distinction should be clearly communicated during consultation using visual aids, before-and-after images of similar body types, and transparent discussion of the procedure’s limitations. While HA fillers can create significant aesthetic improvements, they do not substitute for fat reduction or muscular hypertrophy. Clear communication builds trust and aligns outcomes with realistic expectations.

Increased adipose mobility introduces a risk of filler migration or displacement, particularly in dynamic regions such as the arms and chest. To mitigate this, the use of blunt cannulas and highly cross-linked, high G-prime HA fillers is recommended for better stability, tissue integration, and durability in softer tissues.

Another key consideration is cost-effectiveness. Achieving optimal results in higher body fat individuals often requires larger filler volumes, significantly raising treatment costs. For instance, increasing the pectoral injection volume from 30 mL to 35 mL per side entails a proportional rise in material usage and financial burden. As such, practitioners should ensure patients are informed of the likely cost range during initial consultation.

While not a replacement for lifestyle modification, these procedures may offer psychological benefits by providing immediate aesthetic gratification. This visual enhancement can act as a motivational catalyst, encouraging patients to adopt healthier habits such as consistent exercise and improved diet—creating a potential feedback loop that extends benefits beyond aesthetics.^6^ However, it remains essential for practitioners to ensure that patients seek treatment for appropriate reasons and do not harbor unrealistic expectations or underlying body image disorders. A holistic, ethically grounded approach ensures both clinical success and patient well-being.

Compared to fat grafting, implants, and non-invasive technologies such as cryolipolysis and high-intensity focused electromagnetic (HIFEM) devices (e.g., EMSculpt), hyaluronic acid (HA) fillers offer a distinct balance of safety, reversibility, and procedural simplicity. Fat grafting requires liposuction, anesthesia, and surgical preparation, with variable graft survival and the potential for nodularity or asymmetry. Implants, while effective for volume enhancement, involve invasive surgery, longer recovery, and risks such as capsular contracture, infection, or implant migration. Despite the growing use of hyaluronic acid fillers for body contouring, there is a lack of evidence-based guidelines on hyaluronidase dosing and efficacy for managing complications in non-facial regions.^7^

This study is subject to several limitations. First, it represents a single-case report, which inherently limits generalizability and precludes statistical analysis; as such, the findings should be viewed as preliminary and hypothesis-generating. Second, the absence of a control group restricts the ability to attribute observed outcomes solely to the intervention. Future controlled trials are warranted to validate the efficacy of this technique. Third, outcome evaluation relied on subjective patient-reported satisfaction scores, which, while clinically relevant, lack objective quantification. Incorporating tools such as 3D imaging or standardized aesthetic scoring systems in future studies would strengthen the evidence base. Fourth, the follow-up duration was limited to one month; longer-term assessment through 6 months is ongoing to evaluate durability, safety, and potential complications.

## Conclusion

The refined injection protocols presented in this case highlight a customized approach to upper body contouring in individuals with higher body fat percentages—an often underrepresented demographic in aesthetic medicine. By addressing the challenges posed by subcutaneous adiposity through strategic layering, dynamic assessment, and feathering techniques, practitioners can achieve subtle yet impactful enhancements. This method offers a safe, reversible, and patient-centered alternative for individuals seeking improved body contours without surgery, ultimately promoting higher satisfaction and aesthetic confidence.

## Ethics statement

This study was conducted in accordance with the principles of the Declaration of Helsinki. Given the nature of a single-patient case report, written informed consent was obtained from the patient for both the procedure and the publication of associated clinical data and images.

## Conflict of interest

The authors declared no potential conflicts of interest with respect to the research, authorship, and publication of this article. This study was conducted in compliance with the principles set forth in the Declaration of Helsinki.

## Funding

The authors received no financial support for the research, authorship, and publication of this article. The products utilized in this study were donated by the injectors for the purposes of this study.

## Author contributions

*Conceptualization*: Kyu-Ho Yi, Jovian Wan,Song Eun Yoon, Lucas Basmage, Carlos Bautzer. *Writing—Original Draft Preparation*: Jovian Wan, Kyu-Ho Yi. *Writing—Review and Editing*: Kyu-Ho Yi, Jovian Wan, Song Eun Yoon, Lucas Basmage, Carlos Bautzer. *Visualization*: Kyu-Ho Yi, Jovian Wan, Song Eun Yoon, Lucas Basmage, Carlos Bautzer. *Supervision*: Kyu-Ho Yi. All authors have reviewed and approved the article for submission.
